# Natural evolution of ductus arteriosus with noninterventional conservative management in extremely preterm infants born at 23-28 weeks of gestation

**DOI:** 10.1371/journal.pone.0212256

**Published:** 2019-02-13

**Authors:** Se In Sung, Yun Sil Chang, Jisook Kim, Jin Hwa Choi, So Yoon Ahn, Won Soon Park

**Affiliations:** Department of Pediatrics, Samsung Medical Center, Sungkyunkwan University School of Medicine, Seoul, Korea; Centre Hospitalier Universitaire Vaudois, FRANCE

## Abstract

This study aimed to determine the natural course of patent ductus arteriosus (PDA) with noninterventional conservative management and whether the presence and/or prolonged duration of hemodynamically significant (HS) PDA increased the risk of mortality and morbidities in extremely preterm (EPT) infants. We retrospectively reviewed the medical records of EPT infants born at 23–28 weeks of gestation (n = 195) from January 2011 to June 2014, when PDA was managed with noninterventional conservative treatment. We stratified infants into three subgroups of 23–24, 25–26, and 27–28 weeks and analyzed the prevalence and natural evolution of HS PDA, defined as ventilator dependency and PDA size ≥2 mm. Multivariate regression analyses determined if the presence and/or prolonged duration of HS PDA increased the risk for mortality and/or morbidities. The overall incidence of HS PDA was 57% (111/195) at the end of the first postnatal week. In subgroup analyses, infants with 23–24 weeks of gestation had the highest incidence (93%, 50/54), with 64% (47/74) for 25–26 weeks and 21% (14/67) for 27–28 weeks. Six (5%) of 111 infants with HS PDA were discharged without ductus closure, 4 had spontaneous PDA closure on follow up, and device closure was performed for 2 infants. In the multivariate analyses, the presence or prolonged duration (per week) of HS PDA was not associated with the risk of mortality and/or morbidities. Spontaneous closure of HS PDA was mostly achieved, even in EPT infants, with a noninterventional conservative approach. In conclusion, our data showed the incidence and natural course of HS PDA in EPT infants and suggested that the presence or prolonged duration of HS PDA might not increase the rate of mortality or morbidities.

## Introduction

Persistent patent ductus arteriosus (PDA) in premature infants is associated with increased mortality and morbidities including bronchopulmonary dysplasia (BPD), necrotizing enterocolitis (NEC), and intraventricular hemorrhage (IVH), but evidence supporting their causal relationships is lacking [1–4]. A deeply ingrained hypothesis is that left-to-right shunt is a direct cause of adverse outcomes in preterm infants with persistent PDA; thus, developing sequelae are a function of the magnitude and duration of left-to-right ductal shunting [1,6]. This hypothesis has suggested mandatory closure of PDA at the earliest time with medical and/or surgical therapy for persistent PDA in preterm infants [5,6]. However, compelling evidence is lacking on the therapeutic efficacy and safety of conventional medical or surgical therapy of PDA from meta-analyses and a few small prospective studies [7,8]. A high spontaneous closure rate for PDA, even in extremely low birth-weight (ELBW) infants, has caused a trend in PDA treatment toward more permissive and less aggressive conservative approaches [7–11].

Few studies have chronologically monitored the natural course of persistent PDA with a noninterventional approach, especially in extremely preterm (EPT) infants, because of early administration of medical and/or surgical treatment targeting closure [10,12]. Previously, we demonstrated that a noninterventional approach to hemodynamically significant (HS) PDA is associated with significantly less BPD compared to mandatory closure in EPT infants with 23–26 weeks of gestation [13]. The primary aim of the present study was to present the natural course of HS PDA before and after hospital discharge until closure in a retrospective cohort of EPT infants born at 23–28 weeks of gestation who underwent noninterventional PDA management. Since we focused on the natural course of HS PDA in infants who exclusively received conservative management, we could investigate the temporal change of HS PDA without interference of the effective active PDA treatment. The secondary aim of this study was to compare mortality and selected neonatal morbidities between patients with and without HS PDA and to determine if prolonged duration of HS PDA had an adverse effect on the risk of mortality and morbidities.

## Methods

**Fig 1:**
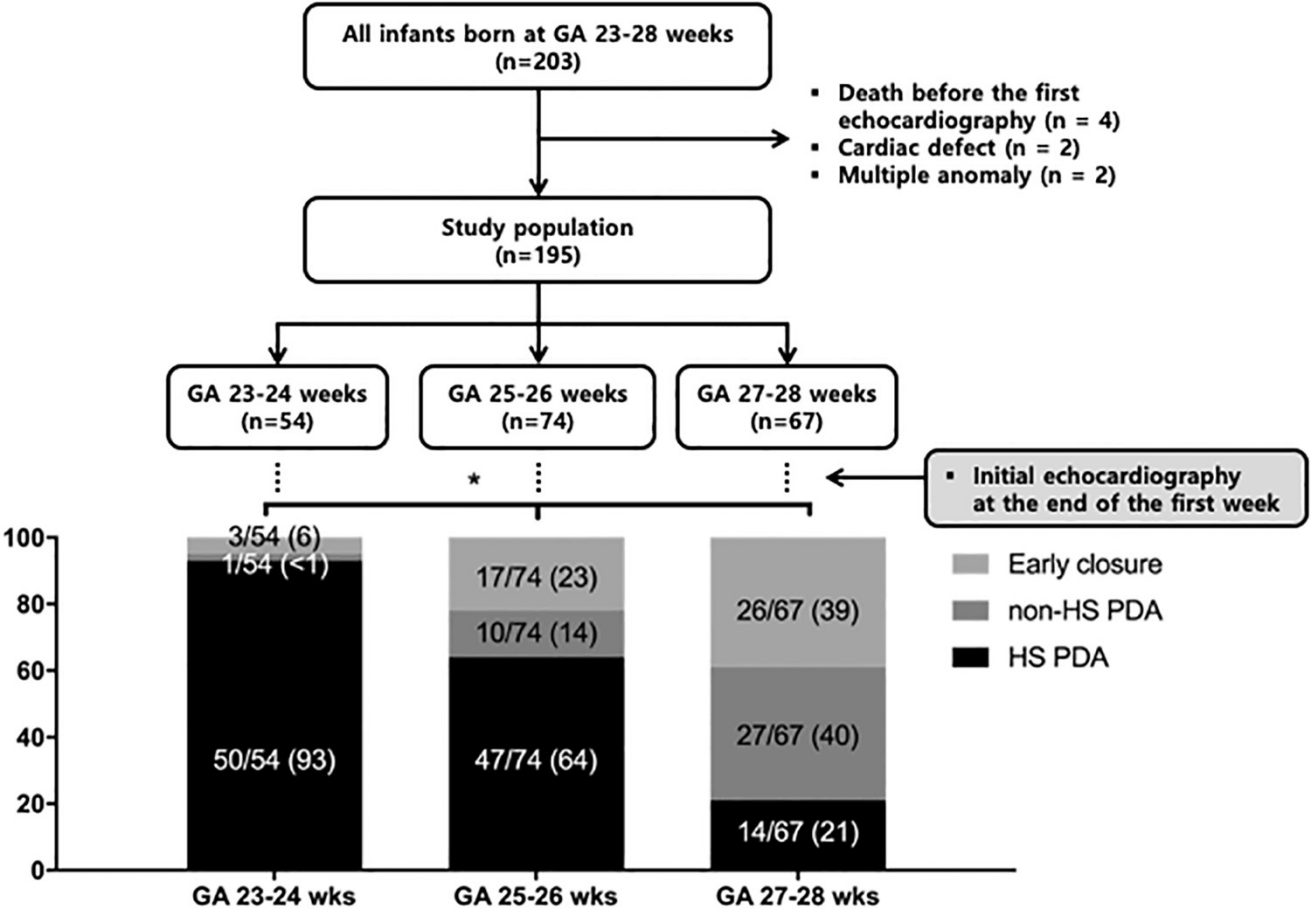
Initial incidence of HS-PDA, HIS-PDA, and early closure at the end of the first postnatal week according to gestational age subgroup. GA, gestational age; HS-PDA, hemodynamically significant patent ductus arteriosus; HIS-PDA, hemodynamically insignificant patent ductus arteriosus.

Data collection was approved by the Institutional Review Board of Samsung Medical Center (SMC) with waiver of informed consent for the retrospective chart review (IRB No. SMC 2016-10-045). The medical records of 203 EPT infants born at 23–28 weeks of gestation who were admitted to the SMC NICU from January 2011 to June 2014 were reviewed retrospectively (Fig 1). After excluding infants who died before the first echocardiography and those who had congenital heart disease or multiple congenital anomalies, 195 EPT infants were included for analysis. We stratified the EPT infants into 23–24, 25–26, and 27–28 weeks of gestation and compared mortality and morbidities such as BPD, NEC, and IVH according to the presence or absence and duration of HS PDA. HS PDA was defined as ≥2 mm with predominant left-to-right shunt on echocardiography (ACUSON SeQuoia C512; Siemens Medical Solutions, Mountain View, California, USA) initially performed at the end of the first postnatal week and need for ventilator support with symptoms and signs suggestive of symptomatic PDA including cardiac murmur, hypotension, widened pulse pressure, or respiratory deterioration. The initial echocardiography was deferred until the end of the first week because early spontaneous ductal closure could occur during the first week even in EPT infants [10]. Non-HS PDA was defined as PDA <2 mm on echocardiography, or there was no requirement for ventilator support regardless of PDA size. Follow up echocardiography was conducted if any symptoms/signs suggestive of PDA occurred and regularly at 2- to 4-week intervals until PDA closure.

During the study period, all infants with any PDA were managed with noninterventional conservative treatment without any rescue treatment. Judicious fluid restriction was maintained for the first two months of life. The target fluid volume was individualized for each infant after clinical evaluation of volume overload including body weight change, hyponatremia, or cardiomegaly. When the condition of the EPT infants with HS PDA deteriorated clinically with symptoms and signs of HS PDA, the attending neonatologist evaluated whether the deteriorated condition was due to HS PDA and considered rescue treatment for PDA, including surgical ligation.

Clinical characteristics analyzed were gestational age (GA), birth weight, Apgar scores at 1 and 5 min, sex, small for gestational age, and chorioamnionitis. GA was determined by maternal last menstrual period and modified Ballard test. Small for gestational age was defined as birth weight <10th percentile. Chorioamnionitis was confirmed by placental pathology. Oliguric renal failure was defined as urine output <0.5 ml/kg/day for ≥24 hours combined with serum creatinine ≥2.0 mg/dl. Nonoliguric renal dysfunction was defined as serum creatinine ≥2.0 mg/dl without oliguria.

Outcome measures including mortality, BPD defined as need for ≥ supplemental oxygen at 36 weeks of gestation [14], IVH (grade 2, grade ≥3) [15], periventricular leukomalacia, NEC (Bell stage ≥ IIb) [16], and retinopathy of prematurity (ROP) (stage 2, ≥3) [17]. Data on long-term neurodevelopmental outcomes were not collected.

To demonstrate the natural time course of HS PDA according to gestational age, cumulative incidence rates for ductal patency at 23–24, 25–26, and 27–28 weeks of gestation were analyzed. Multivariate regression analyses were used to calculate adjusted odds ratios for mortality and morbidities of BPD, IVH (grade ≥3), NEC (Bell stage ≥IIb), and ROP (stage ≥3) with 95% confidence intervals according to the presence or prolonged duration (per increase of 7 days) of HS PDA.

### Statistical analyses

**Fig 2:**
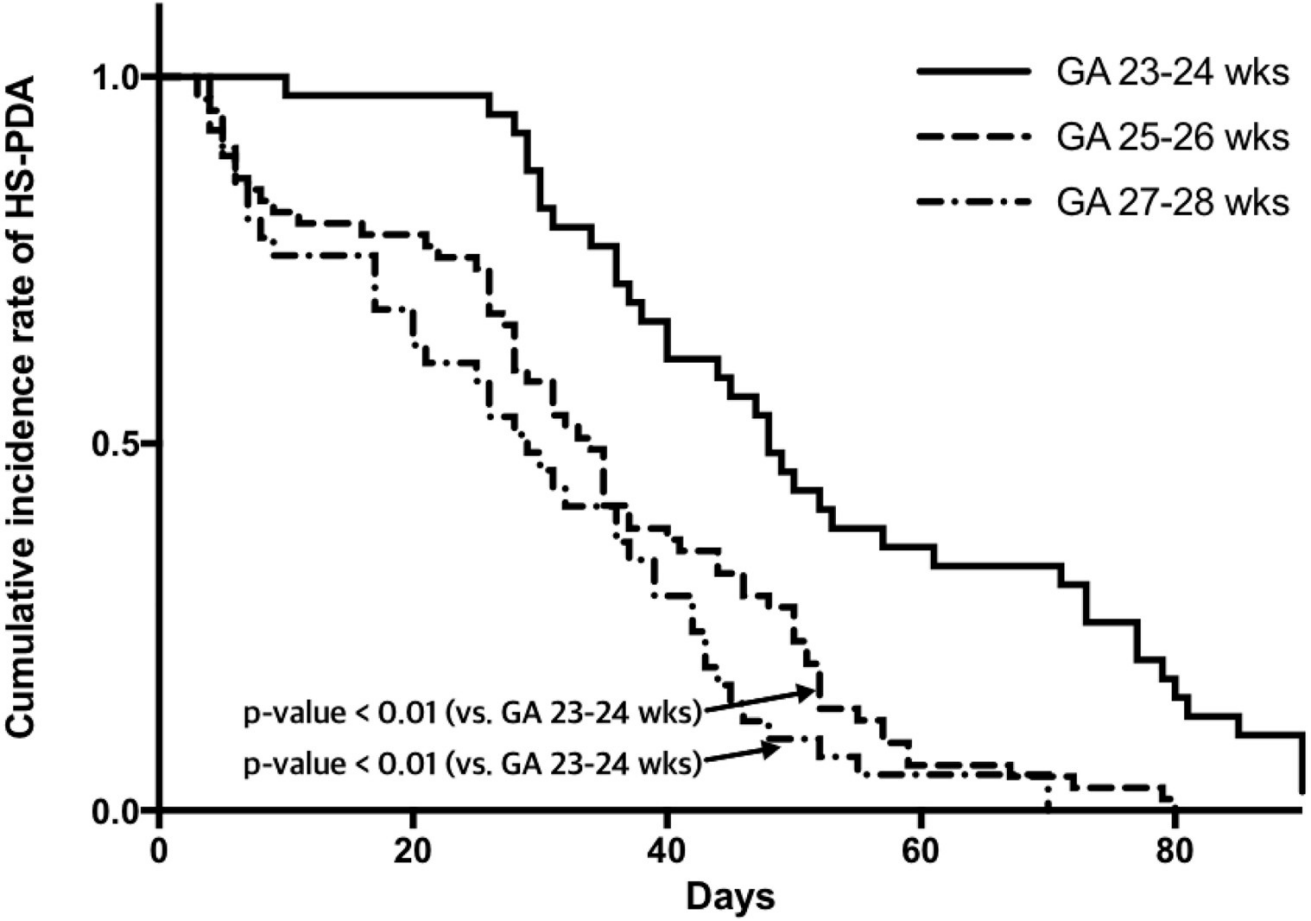
Cumulative incidence rate of ductal patency during hospitalization in infants with initial HS PDA according to gestational age group. PDA, patent ductus arteriosus; GA, gestational age.

Statistical differences between infants with and without HS PDA were tested by χ^2^ tests for nominal variables and *t*-tests or Mann-Whitney *U* tests for continuous variables. Cumulative incidence rates of PDA were demonstrated according to GA group (Fig 2) using Kaplan-Meier estimations, and differences were compared using Cox proportional-hazards regression. Multivariable analysis using binary logistic regression was performed to calculate adjusted odds ratios for risk of neonatal outcomes by presence of HS PDA in infants with any PDA. The impact of ductal patency duration was also tested using multivariable analysis in infants with HS PDA. A p value < 0.05 was considered statistically significant. Statistical analysis was performed using STATA 13.0 software (STATACorp LP, College Station, TX, USA) and R 3.0.3 (Vienna, Austria; http://www.R-project.org).

## Results

### Natural course of PDA in EPT infants

Among infants with a GA of 23–28 weeks born during the study period, 8 infants were excluded because of death before first echocardiography (n = 4), congenital heart disease (n = 2), and multiple congenital anomalies (n = 2). We included 195 EPT infants for further analyses. According to echocardiography initially performed at the end of the first postnatal week, among 195 EPT infants, 111 (57%) had HS PDA, 38 (19%) had non-HS PDA, and 46 (24%) had early closure of PDA (Fig 1). In subgroup analyses, the prevalence rate of HS PDA was GA dependent, showing the highest rate of 93% (50/54) in infants with 23–24 weeks of gestation, followed by 64% (47/74) with 25–26 weeks and the lowest rate of 21% (14/67) with 27–28 weeks.

For 111 infants with HS PDA, the size of the PDA was similar between subgroups. The mean postnatal age of PDA closure during hospitalization at postnatal days (P) of 53 in infants with 23–24 weeks of gestation was significantly delayed compared with P 41 for infants with 25–26 weeks of gestation or P 36 for 27–28 weeks (Fig 2, Table 1). Six (5%) of 111 infants with HS PDA were discharged home without closure. On follow up, 4 infants had spontaneous ductus closure on echocardiography at 4, 4, 5, and 9 months of postnatal age. In the remaining 2 infants, PDA was closed by transcatheter occlusion at 12 and 13 months of age. The 38 infants with non-HS PDA underwent spontaneous closure during hospitalization at a postnatal age of 37.7 ± 13.7 days (minimum 14 days, maximum 80 days). Although rescue treatment was planned for back-up intervention including surgical ligation, no infants with or without HS PDA actually received the rescue treatment during hospitalization.

**Table 1:** Characteristics of HS PDA according to gestational age subgroup.

	Total (n = 111)			
	GA 23–24 weeks (n = 50)	GA 25–26 weeks (n = 47)	GA 27–28 weeks (n = 14)	Total (n = 111)
First diagnosis of PDA, day	7.6 ± 1.7	6.1 ± 2.0	6.8 ± 4.8	6.4 ± 2.6
PDA size at time of first diagnosis, mm	2.5 ± 0.5	2.6 ± 0.5	2.4 ± 0.5	2.5 ± 0.6
Age at PDA closure, days (min–max)	53 ± 26 (24–200)	41 ± 15 (11–79)*	36 ± 18 (7–70)*	49 ± 31
*PDA open until discharge*				
n (%)	3 (6)	2 (4)	1 (7)	6 (5)
Spontaneous closure during follow-up, n (%)	2 (4)	1 (2)	1 (7)	4 (4)
Age at spontaneous closure, months	5, 9	4	4	-
Age at device closure, months	13	12	NA	-

HS, hemodynamically significant

### Clinical characteristics

Demographic and clinical findings in EPT infants according to the presence of HS PDA are shown in S1 Table. The HS PDA (-) group included non-HS PDA and early spontaneous ductal closure. While total GA, birth weight, and Apgar score at 1 and 5 min in infants without HS PDA were significantly higher than those in infants with HS PDA (p value of < 0.01 for each variables), no significant differences in GA and birth weight were observed between GA-matched subgroups of EPT infants with or without HS PDA. The Apgar score at 1 and 5 min was lower in infants with HS PDA (p value <0.01 and 0.01, respectively). The incidence of SGA in infants with a GA of 27–28 weeks with HS PDA was significantly higher than that in GA-matched infants without HS PDA (p value = 0.02).

### Fluid and energy intake, and renal function

Initial mean fluid intake at P 1 of 67 ml/kg/day was gradually increased to 116 ml/kg/day for infants with HS PDA and 122 ml/kg/day for infants without HS PDA. No significant differences in fluid intake were observed between the two groups from P1 to P 28 (S2 Table). Energy intake, renal function, and use of diuretic drugs were not significantly different between infants with or without HS PDA.

Although infants with and without HS PDA showed similar levels of peak serum creatinine during the first 2 weeks of life, infants with HS PDA had higher risk of oliguric renal failure (12% versus 2%, p value = 0.02) and nonoliguric renal dysfunction (19% versus 2%, p value < 0.01) compared with infants without HS-PDA.

### Adverse outcomes

The incidence of adverse outcomes such as mortality during hospitalization and morbidities of BPD, IVH (≥3), NEC (≥2b), ROP (≥3) and sepsis was not significantly different between infants with or without HS PDA (S3 Table). There was no difference on the growth at the time of hospital discharge between infants with or without HS PDA.

### Adjusted odds ratios for risk of adverse outcomes

In multivariate analyses, adjusted odds ratios for risk of adverse outcomes such as mortality during hospitalization and the investigated morbidities were not significantly increased by the presence of HS PDA (Table 2). The adjusted odds for developing mortality or the morbidities were not significantly increased by prolonged duration (per week) of HS PDA (Table 3).

**Table 2:** Adjusted odds ratios for risk of adverse outcomes by presence of HS PDA.

Outcome	Adjusted OR* (95% CI)	p value
Mortality during hospitalization	0.74 (0.25–2.22)	0.59
Intraventricular hemorrhage grade III-IV	1.89 (0.53–6.77)	0.33
Retinopathy of prematurity (≥ stage 3)	0.88 (0.25–3.11)	0.84
Bronchopulmonary dysplasia	1.57 (0.70–3.53)	0.27
Necrotizing enterocolitis (≥ stage IIb)	1.24 (0.39–3.98)	0.72
Blood culture-proven sepsis	1.27 (0.55–2.93)	0.58

OR, odds ratio; CI, confidence interval

*adjusted for gestational age, birth weight, small for gestational age, antenatal steroid use

**Table 3:** Adjusted odds ratios for risk of adverse outcomes by duration (per week) of HS PDA.

Outcome	Adjusted OR* (95% CI)	p value
Mortality during hospitalization	0.99 (0.97–1.01)	0.34
Intraventricular hemorrhage grade III-IV	1.07 (0.83–1.39)	0.59
Retinopathy of prematurity (≥ stage 3)	1.01 (0.83–1.24)	0.91
Bronchopulmonary dysplasia	1.27 (0.96–1.68)	0.09
Necrotizing enterocolitis (≥ stage IIb)	0.91 (0.37–2.24)	0.83
Blood culture-proven sepsis	0.99 (0.79–1.24)	0.95

OR, odds ratio; CI, confidence interval

*adjusted for gestational age, birth weight, small for gestational age, antenatal steroid use

## Discussion

The natural evolution of PDA, especially in EPT infants, has not been studied well because PDA is often treated. Our data illustrated the natural course of PDA using a retrospective cohort of EPT infants who all received noninterventional conservative management. HS PDA was observed only in 57% of patients at the end of the first postnatal week. Spontaneous closure of PDA was achieved in 97% (189/195) before hospital discharge and in an additional 2% (4/195) during follow up at the outpatient clinic. Device closure was conducted only in 1% (2/195) of EPT infants with 23–28 weeks of gestation. In concordance with our data, Semberova et al. reported spontaneous closure of PDA in 85% of VLBW infants who underwent conservative PDA management with no medical and/or surgical intervention [18]. Rolland et al. observed a spontaneous closure rate of 73% in EPT infants born before 28 weeks, without any specific treatment to close PDA [12]. Collectively, these findings suggest that spontaneous closure of PDA, even in EPT infants near the limit of viability, can be achieved with noninterventional conservative management. These findings thus suggest that exposure to the risks of therapeutic interventions for ductal closure, even in EPT infants, might not be warranted.

Large but not small left-to-right PDA shunt causes significant hemodynamic consequences including decreased systemic blood pressure and blood flow to organs, increased pulmonary blood pressure and flow, lung edema, and decreased lung compliance [19–22]. However, whether a large PDA causes increased mortality and/or any specific neonatal morbidities remains uncertain. Several clinical studies have identified preterm infants with large PDA shunt volumes using a composite PDA scoring system. Despite lack of proof about their direct cause-and-effect relationship, significant association between a large PDA shunt volume and morbidities such as BPD are observed in these studies [23–26]. Contradictory to these results, in our study, the presence of HS PDA, defined as ≥2 mm with predominant left-to-right shunt on echocardiography only in EPT infants receiving ventilator support with symptoms and signs suggestive of symptomatic PDA (cardiac murmur, hypotension, widened pulse pressure, and respiratory deterioration), was not significantly associated with increased risk of mortality and morbidities. Morbidities were severe IVH, BPD, NEC, sepsis, and ROP; in multivariate analyses, comparisons were relative to infants without HS PDA. Therefore, the development of new and effective diagnostic tools including clinical, biochemical, and echocardiographic findings is necessary to better identify infants with HS PDA who require specific medical and/or surgical treatment.

In addition to the magnitude, the long duration of a large PDA shunt might be an important cause of morbidities. Schena et al. demonstrated that infants with <28 weeks of gestation with hemodynamically significant E3 and/or E4 PDA (based on a scoring system proposed by McNamara and Sehgal) had an added risk of 1.7-times every week for development of BPD, but small, nonsignificant PDA did not [26,27]. In contrast, in our study, the mean HS PDA closure date of P 53 in EPT infants of 23–24 weeks gestation was significantly longer than the P 41 for infants with 25–26 weeks of gestation and P 36 for 27–28 weeks gestation. Prolonged duration per week of HS PDA was not associated with increased mortality and/or morbidities, including BPD, in multivariate analyses. In concordance with our data, our previous study showed significantly lower incidence of BPD despite significantly delayed closure of HS PDA at mean P 44 with the noninterventional approach compared with earlier primary surgical mandatory closure at mean P 13 [13]. However, the detrimental effects of surgical ligation might outweigh the beneficial effects of earlier PDA closure in our previous study. Therefore, our previous and present retrospective data show a favorable outcome for noninterventional conservative management for HS PDA in EPT infants, at least supporting the safety and feasibility of the noninterventional approach [13]. We are currently conducting a prospective double-blind randomized controlled clinical trial comparing the therapeutic efficacy of exclusive pharmacologic treatment with oral ibuprofen vs. placebo therapy (NCT0212819) to determine whether pharmacologic therapy shortens exposure time to HS PDA and whether this could improve mortality and morbidities associated with PDA.

The reasons for lack of increased mortality and/or morbidities despite prolonged exposure with noninterventional conservative management might be attributable to judicious fluid therapy during the first 4 weeks of life. In our previous and present studies, low fluid intake starting from a mean 67 ml/kg/day at P1 with high ambient humidification and increasing to <116 ml/kg during the first 4 weeks of life was achieved without restricting caloric intake or increasing the incidence of electrolyte and renal dysfunctions [13]. Considering the greater and delayed peak of serum creatinine level during the first few postnatal weeks in EPT infants, indicative of low glomerular filtration, the success of the noninterventional conservative approach for HS PDA might depend on maintaining low fluid intake goals to avoid fluid overload for the first 4 weeks of life [28,29]. Liebowitz and Clyman reported higher 67% incidence of moderate-to-large PDA compared to our data of 57% and later development of increased BPD/death by conservative treatment with an initial mean fluid intake of 166 ml/kg/day at P1 and 2 [30]. Semberova et al reported that, in addition to a later mean closure date than our data showed for infants <26 weeks GA, 15% of VLBW infants who received liberal fluid intake were discharged home with open PDA. This finding was much higher than our data showing only 3% of EPT infants discharged home with open PDA [18]. Taken together, these findings suggest that meticulous fluid therapy to avoid excessive fluid intake might be essential to reduce the prevalence of HS PDA and to enhance its earlier closure, reducing associated mortality and/or morbidities [31].

Limitations of this study include the retrospective nature of a non-controlled observational study in a single center and a small number of infants in the subgroups of 23–24 weeks of gestation without HS PDA (n = 4) and 27–28 weeks of gestation with HS PDA (n = 11). A wide and heterogeneous interval between follow-up echocardiography sessions (every 2 to 4 weeks) might not allow for accurate comparisons of the timings of spontaneous closure among the groups. We acknowledge the lack of long-term growth and neurodevelopmental outcome data. An ongoing prospective double-blind randomized clinical trial will clarify these issues (NCT0212819). Arbitrary criteria for HS PDA defined as PDA size ≥2 mm on echocardiography with predominant left-to-right shunt only in intubated and mechanically ventilated infants with symptoms/signs of PDA and/or respiratory deterioration could be another limitation. Detailed information of the echocardiographic findings indicating volume overload of the left heart was unavailable and was not adjusted for body size in this study. Although none of the EPT infants received any intervention regardless of the presence of HS or non-HS PDA in this study, we identified infants with HS PDA in a strict manner to determine the natural course of HS PDA and to avoid the chance of selection bias favoring non-HS PDA infants with a less severe condition [26,32]. We chose to evaluate PDA at 1 week of age to avoid the confounding bias caused by the high 30–40% closure rate of PDA within the first week of life [10,32–33]. We suggest the need to develop more detailed and accurate definitions of HS PDA to better identify high-risk infants who might benefit from therapeutic intervention.

In conclusion, the presence of HS PDA defined as ≥2 mm in size and ventilator dependency did not increase mortality or associated morbidities such as BPD, IVH, and NEC. Although natural closure of HS PDA was significantly delayed in infants with 23–24 weeks of gestation compared with infants with 25–28 weeks of gestation, no significant increase in mortality and/or morbidities was observed in infants with HS PDA with a noninterventional conservative approach with judicious and meticulous fluid restriction without medial and/or surgical therapy. These findings warrant a future prospective randomized study to determine the potential short-term and long-term benefits and risks of noninterventional conventional management of HS PDA in EPT infants.

## Supporting information

S1 Table Demographic characteristics in infants with or without HS PDA according to gestational age subgroup. HS, hemodynamically significant; PDA, patent ductus arteriosus; GA, gestational age; *p value < 0.05 versus HS PDA (+).
(DOCX)

S2 Table Fluid and energy intake and renal function in infants with or without HS PDA. Oliguric renal failure (urine output < 0.5 mL/kg/ day for ≥24 hours + serum creatinine >2.0 mg/dL); nonoliguric renal dysfunction (no oliguria + serum creatinine >2.0 mg/dL); diuretic use (≥3 days during the first 2 weeks of life); *p value < 0.05 versus HS PDA (+).
(DOCX)

S3 Table Adverse outcomes of infants with or without HS PDA according to gestational age subgroup. HS, hemodynamically significant; PDA, patent ductus arteriosus; GA, gestational age, * p value < 0.05 versus HS-PDA (+).
(DOCX)

## Abbreviations

BPD: bronchopulmonary dysplasia
EC: early closure within 2 weeks
GA: gestational age
HIS: hemodynamically insignificant
HS: hemodynamically significant
NICU: neonatal intensive care unit
PDA: patent ductus arteriosus
VPI: very preterm infant
