# Supplementary material for: Natural evolution of ductus arteriosus with noninterventional conservative management in extremely preterm infants born at 23-28 weeks of gestation
Source: PLoS One. 2019 Feb 13;14(2):e0212256. doi: 10.1371/journal.pone.0212256 (PMC6374019; doi:10.1371/journal.pone.0212256)
Supplement: S1 Table — HS, hemodynamically significant; PDA, patent ductus arteriosus; GA, gestational age; *p value < 0.05 versus HS PDA (+). (DOCX) [file pone.0212256.s001.docx]

**S1 Table. Demographic characteristics in infants with or without HS PDA according to gestational age subgroup.**

|  | **GA 23-24 weeks (n = 54)** | |  | **GA 25-26 weeks (n = 74)** | |  | **GA 27-28 weeks (n = 67)** | |  | **Total (n = 195)** | |  |
| --- | --- | --- | --- | --- | --- | --- | --- | --- | --- | --- | --- | --- |
|  | **HS-PDA (+)**  **(n = 50)** | **HS-PDA (-)**  **(n = 4)** | **P-value** | **HS-PDA (+)**  **(n = 47)** | **HS-PDA (-)**  **(n = 27)** | **P-value** | **HS-PDA (+)**  **(n = 14)** | **HS-PDA (-)**  **(n = 53)** | **P-value** | **HS-PDA (+)**  **(n = 111)** | **HS-PDA (-)**  **(n = 84)** | **P-value** |
| Gestational age, weeks | 23.6 ± 0.5 | 23.5 ± 0.6 | 0.53 | 25.4 ± 0.5 | 25.6 ± 0.5 | 0.08 | 27.2 ± 0.4 | 27.5 ± .05 | 0.07 | 24.8 ± 1.3 | 26.7 ± 1.2^*^ | <0.01 |
| Birth weight, g | 648 ± 83 | 572 ± 158 | 0.11 | 791 ± 145 | 890 ± 164 | 0.15 | 989 ± 411 | 1024 ± 211 | 0.67 | 751 ± 211 | 959 ± 221^*^ | <0.01 |
| Male, n (%) | 26 (52) | 3 (75) | 0.38 | 28 (60) | 14 (52) | 0.52 | 10 (71) | 28 (53) | 0.03^*^ | 64 (58) | 45 (54) | 0.41 |
| Small for gestational age, n (%) | 3 (6) | 1 (25) | 0.16 | 5 (11) | 2 (7) | 0.65 | 6 (43) | 8 (15) ^*^ | 0.02^*^ | 14 (13) | 11 (13) | 0.92 |
| Cesarean section, n (%) | 35 (70) | 2 (50) | 0.41 | 41 (87) | 22 (81) | 0.50 | 9 (64) | 46 (87) | 0.05 | 85 (77) | 70 (83) | 0.25 |
| Antenatal steroid, n (%) | 40 (80) | 3 (75) | 0.81 | 39 (83) | 24 (89) | 0.49 | 13 (93) | 52 (98) | 0.30 | 93 (83) | 79 (94) | 0.05 |
| Chorioamnionitis, n (%) | 31 (62) | 2 (50) | 0.64 | 25 (53) | 21 (78) | 0.04^*^ | 6 (43) | 30 (57) | 0.36 | 62 (56) | 53 (63) | 0.31 |
| Apgar score, 1 min | 4.1 ± 1.1 | 3.3 ± 1.0 | 0.16 | 4.7 ± 1.5 | 5.9 ± 1.7 | 0.01^*^ | 4.6 ± 1.5 | 5.2 ± 1.9 | 0.32 | 4.4 ± 1.4 | 5.3 ± 1.9^*^ | <0.01^*^ |
| Apgar score, 5 min | 6.5 ± 1.3 | 5.5 ± 1.0 | 0.12 | 6.8 ± 1.3 | 7.7 ± 1.0 | 0.01^*^ | 7.5 ± 1.2 | 7.3 ± 1.8 | 0.94 | 6.8 ±1.4 | 7.4 ± 1.6^*^ | 0.01^*^ |

HS, hemodynamically significant; PDA, patent ductus arteriosus; GA, gestational age; ^*^p value < 0.05 versus HS PDA (+)
