# Supplementary material for: Natural evolution of ductus arteriosus with noninterventional conservative management in extremely preterm infants born at 23-28 weeks of gestation
Source: PLoS One. 2019 Feb 13;14(2):e0212256. doi: 10.1371/journal.pone.0212256 (PMC6374019; doi:10.1371/journal.pone.0212256)
Supplement: S2 Table — Oliguric renal failure (urine output < 0.5 mL/kg/ day for ≥24 hours + serum creatinine >2.0 mg/dL); nonoliguric renal dysfunction (no oliguria + serum creatinine >2.0 mg/dL); diuretic use (≥3 days during the first 2 weeks of life); *p value < 0.05 versus HS PDA (+). (DOCX) [file pone.0212256.s002.docx]

**S2 Table. Fluid and energy intake and renal function in infants with or without HS PDA.**

|  | **Total (n = 195)** | |
| --- | --- | --- |
|  | **HS-PDA (+) (n = 111)** | **HS-PDA (-) (n = 84)** |
| Fluid intake, mL/kg/day |  |  |
| DOL 1 | 67 ± 11 | 67 ± 9 |
| DOL 7 | 107 ± 22 | 109 ± 21 |
| DOL 14 | 104 ± 21 | 107 ± 23 |
| DOL 21 | 107 ± 27 | 112 ± 25 |
| DOL 28 | 116 ± 23 | 122 ± 24 |
| Energy intake, kcal/kg/day |  |  |
| DOL 1 | 45 ± 10 | 45 ± 12 |
| DOL 7 | 70 ± 18 | 71 ± 20 |
| DOL 14 | 77 ± 18 | 78 ± 19 |
| DOL 21 | 83 ± 19 | 85 ± 21 |
| DOL 28 | 87 ± 18 | 87 ± 22 |
| Renal function |  |  |
| Oliguric renal failure, n (%) | 13 (12) | 2 (2)^*^ |
| Nonoliguric renal dysfunction, n (%) | 21 (19) | 2 (2)^*^ |
| Use of diuretic drugs, days | 2.2 ± 1.9 | 1.4 ± 0.9 |
| Peak blood urea nitrogen level, mg/dL | 33 ± 10 | 30 ± 12 |
| Peak serum creatinine level, mg/dL | 1.4 ± 0.5 | 1.4 ± 0.5 |

Oliguric renal failure (urine output < 0.5 mL/kg/ day for ≥24 hours + serum creatinine >2.0 mg/dL); nonoliguric renal dysfunction (no oliguria + serum creatinine >2.0 mg/dL); diuretic use (≥3 days during the first 2 weeks of life) ; ^*^p value < 0.05 versus HS PDA (+)
