# Supplementary material for: Natural evolution of ductus arteriosus with noninterventional conservative management in extremely preterm infants born at 23-28 weeks of gestation
Source: PLoS One. 2019 Feb 13;14(2):e0212256. doi: 10.1371/journal.pone.0212256 (PMC6374019; doi:10.1371/journal.pone.0212256)
Supplement: S3 Table — HS, hemodynamically significant; PDA, patent ductus arteriosus; GA, gestational age, * p value < 0.05 versus HS-PDA (+). (DOCX) [file pone.0212256.s003.docx]

**S3 Table. Adverse outcomes of infants with or without HS PDA according to gestational age subgroup.**

|  | **GA 23–24 weeks (n = 54)** | |  | **GA 25–26 weeks (n = 74)** | |  | **GA 27–28 weeks (n = 67)** | |  | **Total (n = 195)** | |  |
| --- | --- | --- | --- | --- | --- | --- | --- | --- | --- | --- | --- | --- |
|  | **HS-PDA (+)**  **(n = 50)** | **HS-PDA (-)**  **(n = 4)** | **P-value** | **HS-PDA (+)**  **(n = 47)** | **HS-PDA (-)**  **(n = 27)** | **P-value** | **HS-PDA (+)**  **(n = 14)** | **HS-PDA (-)**  **(n = 53)** | **P-value** | **HS-PDA (+)**  **(n = 111)** | **HS-PDA (-)**  **(n = 84)** | **P-value** |
| Mortality during hospitalization, n (%) | 7 (14) | 2 (50) | 0.06 | 2 (4) | 0 (0) | 0.28 | 2 (29) | 6 (11) | 0.63 | 11 (10) | 8 (10) | 0.63 |
| Intraventricular hemorrhage grade III-IV, n (%) | 9 (18) | 1 (25) | 0.51 | 3 (6) | 1 (4) | 0.62 | 3 (21) | 3 (6) | 0.12 | 15 (14) | 5 (6) | 0.12 |
| Intraventricular hemorrhage grade II, n (%) | 5 (10) | 0 (0) | 0.51 | 0 (0) | 1 (4) | 0.18 | 0 (0) | 4 (8) | 0.29 | 5 (5) | 5 (6) | 0.65 |
| Retinopathy of prematurity (≥ stage 3), n (%) | 4 (8) | 0 (0) | 0.61 | 3 (6) | 4 (15) | 0.23 | 2 (14) | 3 (6) | 0.85 | 9 (8) | 7 (9) | 0.86 |
| Retinopathy of prematurity (stage 2), n (%) | 15 (30) | 1 (33) | 0.90 | 18 (38) | 10 (37) | 0.91 | 7 (50) | 24 (48) | 0.89 | 40 (36) | 35 (44) | 0.28 |
| Bronchopulmonary dysplasia, n (%) | 20 (43) | 0 (0) | 0.14 | 15 (32) | 9 (33) | 0.90 | 5 (36) | 8 (15) | 0.10 | 40 (37) | 17 (21) | 0.13 |
| Necrotizing enterocolitis (≥ stage IIb), n (%) | 6 (12) | 1 (25) | 0.46 | 6 (13) | 1 (4) | 0.20 | 1 (7) | 5 (10) | 0.46 | 13 (12) | 7 (8) | 0.46 |
| Blood culture proven sepsis, n (%) | 16 (32) | 3 (75) | 0.08 | 12 (26) | 4 (15) | 0.32 | 4 (29) | 8 (16) | 0.10 | 32 (29) | 15 (19) | 0.10 |
| *Growth parameters, z-score* |  |  |  |  |  |  |  |  |  |  |  |  |
| Body weight at discharge | -2.3 ± 1.3 | -1.1 ± 1.2 | 0.07 | -2.3 ± 1.2 | -1.7 ± 1.2 | 0.06 | -2.8 ± 2.1 | -2.1 ± 2.1 | 0.30 | -2.3 ± 1.4 | -1.9 ± 1.8 | 0.08 |
| Height at discharge | -3.8 ± 2.0 | -3.2 ± 1.4 | 0.69 | -2.8 ± 2.7 | -2.6 ± 1.4 | 0.68 | -3.9 ± 4.0 | -2.8 ± 3.3 | 0.28 | -3.4 ± 2.6 | -2.7 ± 2.7 | 0.08 |
| Head circumference at discharge | -2.3 ± 1.7 | -2.9 ± 1.2 | 0.63 | -2.4 ± 2.7 | -1.5 ± 1.1 | 0.14 | -2.8 ± 3.9 | -2.1 ± 5.5 | 0.67 | -2.4 ± 2.4 | -1.9 ± 4.4 | 0.39 |

HS, hemodynamically significant; PDA, patent ductus arteriosus; GA, gestational age, ^*^ p value < 0.05 versus HS-PDA (+)
